# Rural residence is not a risk factor for frequent mental distress: a behavioral risk factor surveillance survey

**DOI:** 10.1186/1471-2458-5-46

**Published:** 2005-05-16

**Authors:** James E Rohrer, Tyrone F Borders, Jimmy Blanton

**Affiliations:** 1 Department of Family and Community Medicine, Texas Tech University Health Sciences Center, 1400 Commonwealth Drive, Amarillo Texas 79106, USA; 2 Department of Health Management and Policy, University of North Texas Health Science Center, School of Public Health, Fort Worth, TX, USA; 3 Texas Department of State Health Services, Center for Health Statistics, Austin, Texas, USA

## Abstract

**Background:**

Residents of rural areas may be at increased risk of mental health problems. If so, public health programs aimed at preventing poor mental health may have to be customized for delivery to rural areas. The purpose of this study was to examine the relationship between residing in a rural area and frequent mental distress, which is one indicator of poor mental health.

**Methods:**

The Behavioral Risk Factor Surveillance System (BRFSS) survey for the state of Texas was the source of information about obesity, demographic characteristics, and frequent mental distress (FMD). FMD was defined as poor self-rated mental health during at least half of the days in the last month. Adjusted odds for FMD were computed for rural and suburban respondents relative to urban respondents.

**Results:**

FMD was found to be independently associated with lower education, being younger, being non-Hispanic, being unmarried, and being female. FMD also was associated with being obese or underweight and suburban residence (relative to metro-central city). FMD was not more common among rural respondents than in the metro-central city.

**Conclusion:**

Rural respondents were not at greater risk of frequent mental distress than urban respondents in this sample. Programs seeking to improve community mental health should target persons with less education and extremes in body weight, along with women and single persons, regardless of whether they live in rural or urban areas.

## Background

The concern for health disparities extends to residents of rural areas, who are recognized to face distance barriers when seeking to access health services. Concerns about disparities apply to mental as well as physical health. Studies addressing rural residence as a risk factor for poor mental health are important to verify the existence of a disparity and to clarify its nature.

Singh and Siahpush studied rural residence as a risk factor for suicide. They reported that suicide risk increases for males with increasing levels of rurality and that the rural-urban differentials are increasing over time. Rural suicide rates also were higher for women but the rural-urban differential was found to be decreasing over time [1].

Recently, national experts have emphasized the recognition of mental health as a public health problem [2-4]. Suicide, though important, may not be the most important focus for a public health approach to mental health. Community health programs typically are targeted at moderately healthy populations, not the seriously ill, because the goal is prevention so as to avoid the need for treatment. Less serious mental health concerns affect a large fraction of the population. The more common mental health problems often are preventable and may be amenable to health promotion programs involving self-help strategies. Population surveys such as the one reported here naturally are more relevant to the less severe, more common mental health issues.

Rural health advocates frequently make the case that rural residents have worse health than residents of urban areas, implying that residing in a rural area is a risk factor for poor health [5]. However, it is also possible that the causes of poor health are the same regardless of where one lives, and thus programs should be targeted not at rural locations but at populations that are afflicted with the genuine risk factors (e.g., poverty, low educational attainment, and age).

The purpose of the study reported here was to investigate the relationship between rural residence and frequent mental distress, which can be described as poor self-rated mental health. This study differs from some earlier studies in that it includes a variable representing rural or urban residence in an analysis of data drawn from the Behavioral Risk Factor Surveillance System (BRFSS). The BRFSS does not ordinarily contain a variable representing rural or urban residence of the respondent. The Texas Department of State Health Services recently created such a variable, permitting comparisons that previously were not possible.

## Methods

Data were obtained from the 2003 Texas Behavioral Risk Factor Surveillance (BRFSS) survey. The Texas Behavioral Risk Factor Surveillance System (BRFSS), sponsored by the Texas Department of State Health Services in partnership with the Centers for Disease Control and Prevention (CDC), is an ongoing random-digit dialed telephone survey that collects information from the non-institutionalized, civilian adult population. BRFSS surveys adhere to the highest scientific standards for telephone researchand pose questions relating to health status, personal health habits, and use of preventive health services. The 2003 survey data were collected over a 12-month period frominterviews with 6,035 Texas residents. The overall cooperation rate, or proportion of individuals actually contacted on the phone who completed the survey, was 70%. The more conservative response rate favored by the Council of American Survey Research Organizations (CASRO), which adjusts for the estimated number of eligible respondents in the samplewho could not be contacted due to technical or other barriers, was 41%.

Missing values were excluded from the analyses if they constituted less than 5% of cases for an independent variable. Missing dummy variables were created for independent variables for which 5% or more of the cases had missing values (household income and body mass index). The data set contained 5,757 observations.

### Measures

The dependent variable was frequent mental distress, which was assessed with the following question: "Now, thinking about your mental health, which includes stress, depression and problems with emotions, for how many days during the past 30 days was your mental health not good?" Frequent mental distress was defined as having 14 or more days of "not good" mental health.

The primary independent variable of interest was whether the individual lived in a metropolitan, suburban, or non-metropolitan/rural area. Many prior studies have categorized the area of residence as metropolitan vs. non-metropolitan. However, a dichotomous classification may mask important differences between residents of highly urban and suburban areas. Thus, we chose a more detailed classification system of metropolitan-central city, metropolitan-suburban, and non-metropolitan areas based on respondents' reports of their county of residence. Metropolitan-central city was defined as an urban core county containing 50,000 or more persons. Metropolitan-suburban was defined as a metropolitan county adjacent to a core metropolitan-central city area. All other counties were considered non-metropolitan or rural.

Additional independent factors included demographics and body mass index. Demographic variables were age category (18–24, 25–34, 35–44, 45–54, 55–64, and 65+), race/ethnicity (non-Hispanic white, Hispanic, black, and other), gender, marital status (single/never married/divorced/widowed vs married/member of a couple), educational status (less than a high school degree, a high school degree, some college, and college graduate), and household income (<$25,000, $25,000 to $75,000, ≥$75,000, and income missing). Body mass index (BMI) categories were defined as obese (BMI ≥ 30), overweight (BMI 25–29.99), normal weight (BMI <25 and ≥ 18.5), underweight (BMI < 18.5), and a BMI missing category.

### Analyses

The percentage of respondents with frequent mental distress (FMD) was calculated by urban/rural residence and all other independent variables. Multivariate logistic regression analyses were conducted to estimate adjusted odds ratios (AORs) for selected risk factors. Data were weighted to reflect the demographic distributions of the sample. Because we were interested in testing whether the predictors of FMD differ between residents of metropolitan-central city, metropolitan-suburban, and non-metropolitan areas, we then stratified the analyses by metropolitan status. STATA 8.0 was used to conduct descriptive and multivariate analyses to account for the complex sampling scheme and population weights [6].

## Results

Frequent mental distress was reported by 9.79% of the respondents. The first column of Table 1 describes respondents' demographic, social, economic, and body mass characteristics. Approximately 62% and 21% of respondents lived in metropolitan-central city and metropolitan-suburban areas, respectively, whereas about 17% resided in non-metropolitan counties. The second column describes the percentage of respondents with frequent mental distress by each selected risk or independent variable. Residents of metropolitan-suburban areas had the highest rate of FMD (11.92%). Those residing in metropolitan-central city and non-metropolitan areas had similar rates of FMD (9.34 and 8.77%). After adjustment for other variables, persons in metropolitan-suburban areas had a significantly higher odds of FMD compared to metropolitan-central city residents (AOR = 1.37). Non-metropolitan residents did not have a significantly different adjusted odds of FMD as compared to individuals residing in metropolitan-central city areas.

**Table 1:** Prevalence of frequent mental distress (FMD) and adjusted odds ratios (AOR) for selected risk factors, State of Texas, 2003, (N=5,757)

Risk variable	Overall %	% with FMD (95% CI)	AOR* (95% CI) *N *= 5,757	*P *value
Residence				
Metropolitan-central city	61.50	9.34 (8.24–10.44)	Reference	
Metropolitan-suburb	21.23	11.92 (9.77–14.07)	1.37 (1.06–1.77)	0.016
Non-metropolitan	17.27	8.77 (6.59–10.96)	0.86 (0.63–1.18)	0.350
				
Age group				
18–24	14.66	11.92 (8.80–15.05)	Reference	
25–34	20.23	6.79 (5.04–8.54)	0.92 (0.62–1.34)	0.635
35–44	21.09	9.26 (6.91–11.60)	1.17 (0.78–1.69)	0.488
45–54	18.08	11.40 (9.32–13.49)	1.26 (0.86–1.85)	0.227
55–64	11.88	10.39 (8.29–12.50)	0.91 (0.59–1.41)	0.675
65+	14.07	8.57 (6.84–10.32)	0.51 (0.33–0.78)	0.002
				
Race/ethnicity				
non-Hispanic white	58.94	9.54 (8.61–11.68)	Reference	
Hispanic	28.91	9.22 (7.51–10.95)	0.56 (0.41–0.75)	<0.001
Non-Hispanic Black	8.20	13.58 (9.47–17.68)	0.90 (0.62–1.31)	0.587
Other	3.95	9.72 (5.20–14.24)	1.15 (0.67–0.98)	0.607
				
Sex				
Female	50.91	12.06 (10.80–13.31)	Reference	
Male	49.09	7.44 (6.16–8.73)	0.60 (0.48–0.76)	<0.001
				
Marital status				
Single/never married	38.65	13.28 (11.58–14.98)	Reference	
Married	61.35	7.61 (6.61–8.61)	0.66 (0.54–0.82)	<0.001
				
Educational status				
< high school degree	17.52	13.61 (10.96–16.26)	Reference	
high school degree	27.44	12.84 (10.77–14.91)	0.80 (0.59–1.10)	0.172
some college	26.51	8.72 (7.20–10.24)	0.53 (0.37–0.74)	<0.001
College graduate	28.53	5.50 (4.41–6.60)	0.38 (0.26–0.55)	<0.001
				
Household income				
<$25,000	30.01	15.79 (13.68–17.90)	Reference	
$25,000 to $75,000,	39.27	7.50 (6.28–8.72)	0.52 (0.40–0.67)	<0.001
≥$75,000	18.85	6.40 (4.75–8.06)	0.49 (0.33–0.71)	<0.001
Missing	11.87	7.58 (5.41–9.75)	0.50 (0.35–0.71)	<0.001
				
Body mass index				
Obese	23.02	12.66 (10.58–14.75)	1.56 (1.19–2.05)	0.001
Overweight	34.61	8.76 (7.31–10.21)	1.22 (0.93–1.60)	0.159
Normal	34.07	8.18 (6.75–9.61)	Reference	
Underweight	2.08	24.38 (15.00–33.76)	3.15 (1.82–5.45)	<0.001
BMI missing	6.22	8.85 (5.42–12.27)	0.88 (0.53–1.44)	0.602

CI = confidence interval.

*Adjusted for all other variables in Table 1. *N *= 5,757 prior to weighting.

Age differences in FMD were revealed as those 65 years and older had a significantly lower adjusted odds of FMD than individuals between 18 and 24 years of age (AOR = 0.51). In regard to race/ethnicity, a higher percentage of Blacks/African Americans (non-Hispanic) reported FMD than non-Hispanic whites, Hispanics, or other non-Hispanic racial groups. However, when adjusting for other factors, only Hispanics' odds of FMD differed from non-Hispanic whites' (AOR = 0.56). Males exhibited a significantly lower adjusted odds of FMD (AOR = 0.60) as compared to females. Married or living-together persons had a significantly lower adjusted odds of FMD than single persons (AOR = 0.66). Educational attainment and household income, two indicators of socioeconomic status, were also associated with FMD. Compared with those with less than a high school degree, individuals with some college education as well as those who were college graduates had lower adjusted odds of FMD (AOR = 0.53 and 0.38, respectively). Similarly, individuals who had higher household incomes experienced a lower adjusted odds of FMD. Body mass index categories of obesity and underweight were associated with substantially higher odds (AORs = 1.56 and 3.15, respectively) of FMD as compared to normal weight persons.

We also performed analyses of the predictors of FMD by metropolitan-central city, metropolitan-suburban, and non-metropolitan status (Table 2). The findings for each model were similar with a few notable exceptions. Among those residing in metropolitan-central city areas, Hispanics had a significantly lower odds (AOR = 0.53, 95% CI = 0.37–0.77) of FMD than non-Hispanic whites. However, among residents of metropolitan-suburban and non-metropolitan areas, the odds of FMD did not differ between Hispanics and non-Hispanic whites. Similarly, male gender was significant in the model for metropolitan-central city residents (AOR = 0.50, 95% CI = 0.37–0.68) but insignificant in the remaining two models. Married or living-together persons were less likely to have FMD among metro-central city respondents, but marital status was not significant elsewhere in the state.

**Table 2:** Adjusted odds ratios (AOR) of frequent mental distress (FMD), stratified by metropolitan status

Risk variable	Metropolitan-Central City *N *= 3,505	*P *value	Metropolitan-Suburban *N *= 1,251	*P *value	Non-Metropolitan *N *= 1,001	*P *value
	AOR * (95% CI)		AOR * (95% CI)		AOR * (95% CI)	
						
Age group						
18–24	Reference		Reference		Reference	
25–34	1.25 (0.77–2.03)	0.366	0.40 (0.19–0.85)	0.016	0.94 (0.32–2.72)	0.904
35–44	1.40 (0.85–2.30)	0.186	0.63 (0.28–1.40)	0.256	1.73 (0.64–4.67)	0.283
45–54	1.42 (0.87–2.30)	0.157	0.96 (0.46–1.98)	0.904	1.49 (0.50–4.46)	0.476
55–64	1.16 (0.65–2.09)	0.614	0.59 (0.25–1.37)	0.218	0.93 (0.31–2.77)	0.892
65+	0.60 (0.33–1.08)	0.091	0.26 (0.11–0.59)	0.001	0.77 (0.29–2.02)	0.586
						
Race/ethnicity						
Non-Hispanic white	Reference		Reference		Reference	
Hispanic	0.53 (0.37–0.77)	0.001	0.49 (0.23–1.03)	0.058	0.88 (0.41–1.88)	0.738
Non-Hispanic Black	0.90 (0.59–1.39)	0.648	0.88 (0.31–2.46)	0.804	0.90 (0.33–2.50)	0.845
Other	1.23 (0.62–2.43)	0.547	0.81 (0.25–2.63)	0.725	1.57 (0.37–6.65)	0.540
						
Sex						
Female	Reference		Reference		Reference	
Male	0.50 (0.37–0.68)	<0.001	0.73 (0.47–1.14)	0.171	0.77 (0.44–1.36)	0.368
						
Marital status						
Single/never married	Reference		Reference		Reference	
Married	0.56 (0.43–0.74)	<0.001	0.81 (0.50–1.30)	0.384	0.98 (0.56–1.70)	0.931
						
Educational status						
< high school degree	Reference		Reference		Reference	
high school degree	0.75 (0.51–1.12)	0.165	0.85 (0.44–1.61)	0.623	0.90 (0.42–1.96)	0.800
some college	0.59 (0.38–0.92)	0.018	0.36 (0.17–0.77)	0.008	0.71 (0.31–1.62)	0.420
College graduate	0.42 (0.26–0.67)	<0.001	0.41 (0.20–0.83)	0.013	0.15 (0.05–0.50)	0.002
						
Household income						
<$25,000	Reference		Reference		Reference	
$25,000–$75,000	0.53 (0.38–0.74)	<0.001	0.56 (0.32–0.97)	0.040	0.34 (0.17–0.71)	0.004
≥$75,000	0.51 (0.33–0.80)	0.003	0.36 (0.16–0.82)	0.016	0.94 (0.38–2.42)	0.905
Income missing	0.45 (0.28–0.71)	0.001	0.62 (0.27–1.39)	0.245	0.41 (0.18–0.96)	0.040
						
Body mass index						
Obese	1.96 (1.37–2.80)	<0.001	1.04 (0.61–1.79)	0.881	1.28 (0.58–2.87)	0.542
Overweight	1.19 (0.82–1.72)	0.357	1.16 (0.70–1.92)	0.562	1.69 (0.78–3.63)	0.181
Normal	Reference		Reference		Reference	
Underweight	2.98 (1.54–5.78)	0.001	1.26 (0.37–4.29)	0.710	11.94 (3.66–38.97)	<0.001
BMI missing	0.22 (0.67–2.23)	0.513	0.35 (0.11–1.16)	0.087	0.74 (0.19–2.83)	0.656

CI = confidence interval.

* Adjusted for all other variables in Table 1.

## Discussion

The theory that residing in a rural location rather than an urban area is a risk factor for frequent mental distress is not supported by our data. Instead, persons living in suburbia were found to be at risk for FMD after adjusting for other risk factors. We suspect that some stressors may be at work in the suburbs that are not found in rural areas or central cities. This warrants further investigation.

In our data, lifestyle variables (obesity and underweight), Hispanic ethnicity, female gender, being unmarried, and having less education are more important risk factors for FMD than rural residence. Because obesity and underweight are related to frequent mental distress, public health programs designed to achieve normal body weight might improve mental health [7]. The potential for population-level health promotion strategies to reduce the prevalence of mental health problems is important, since the medical care system has been chastised for its insensitivity to underlying mental health problems among patients who present with physical symptoms [8-11]. Efforts directed at improving the quality of mental health services delivered in primary care settings have had mixed results [9]. Clearly, this is another case where population-based preventive strategies by public health agencies might be more effective than individualized medical treatment of mental health problems, at least for less severe or incipient cases [7,11].

The widespread and increasing [12-14] prevalence of frequent mental distress in the population (8.6 percent of adults nationally a decade ago and ten percent in 2001 [13]) necessitates a public health response. In addition, the psychological consequences of any future terrorist attacks or natural disasters will require population-based responses that can reach large numbers of people in a short time [15].

## Conclusion

This study examined how rural residence was related to frequent mental distress in the general population in a single state. Our findings are a direct result of the modeling strategy employed. Accordingly, replication of these results by other investigators is important. Limitations of the study include a modest participation rate, the cross-sectional design, and the loss of some cases due to missing data. The cross-sectional nature of the study makes causal conclusions impossible. Furthermore, the use of a self-reported single item to measure frequent mental distress does not equate to providing diagnostic information. Another important limitation is that most seriously mentally ill persons are likely to be missed by a survey such as this because they at increased risk of being homeless or otherwise unavailable for telephone interviews. Nevertheless, the single item has been accepted by public health researchers for use in population surveys [11-14,16-18].

Health status in rural populations is believed to be lower than in urban areas, partly due to higher rates of poverty [5]. This assertion specifically relates to rates of chronic disease, infant mortality, injury rates, trauma mortality, and overall mortality. However, most government sponsored surveys do not include a variable that reflects urban or rural residence. Our study is the first to use a Behavioral Risk Factor Surveillance System (BRFSS) survey with a variable reflecting rural residence. Our results do not support the concern about rural residence being a risk factor for poor mental health. The risk factors for poor mental health are found in urban as well as rural areas, although the delivery of services may be more difficult in rural areas.

When Singh and Siahpush reported that suicide rates were higher in rural areas, they attributed this finding partly to social isolation [1]. If this theory was correct, then we would expect frequent mental distress also to be more prevalent among rural respondents in our data. However, we did not find rural respondents to be at greater risk of frequent mental distress than urban respondents. We question the generalization that rural people experience greater levels of social isolation, since rural communities sometimes may be more integrated and supportive than urban neighborhoods.

Community health programs that are supported by strong evidence will, for the most part, focus on three strategies: policy changes, mass communication, and targeted health education [19]. Applied to risk factors for frequent mental distress, examples of policy changes might be zoning practices that promote physical activity, labeling of unhealthy menu items in restaurants, and increases in liquor and cigarette taxes. Media campaigns could be employed to raise awareness about anxiety, depression, stress, and healthy coping strategies. The third approach to community mental health is to target health education programs at persons who are at high risk. In Texas, this would be the elderly, the poor, and persons who are undereducated. Since none of these population-based strategies involve personal health services or require substantial investments in physical plant, they could each be carried out without regard to whether the targeted populations reside in rural or urban areas.

## Competing interests

The author(s) declare that they have no competing interests.

## Authors' contributions

TFB carried out the statistical analysis, wrote the methods section, and wrote the results section. JB wrote the section on sampling and the BRFSS in Texas. JER conceived of the study, planned the design, and wrote the background, discussion and conclusions.

## Pre-publication history

The pre-publication history for this paper can be accessed here:


